# [2-(1*H*-Indol-4-yl)eth­yl]dipropyl­amine

**DOI:** 10.1107/S1600536812015474

**Published:** 2012-04-21

**Authors:** De-Cai Wang, Hua-Yang Pan, Jun-Song Song, Ping Wei, Ping-Kai Ou-yang

**Affiliations:** a State Key Laboratory of Materials-Oriented Chemical Engineering, School of Pharmaceutical Sciences, Nanjing University of Technology, Xinmofan Road No. 5 Nanjing, Nanjing 210009, People’s Republic of China

## Abstract

In the title compound, C_16_H_24_N_2_, the aliphatic amine substituent is rotated almost orthogonally [C—C—C—C torsion angle = 75.7 (3)°] out of the plane of the indole unit. The amine N atom has a pyramidal configuration deviating by 0.380 (3) Å from the plane of the adjacent C atoms. All of the aliphatic groups are in extended transoid conformations. In the crystal, mol­ecules form chains along the *a* axis *via* N—H⋯N hydrogen bonds.

## Related literature

For the synthesis and applications of the title compound, see: Srivastava *et al.* (1999 ▶).
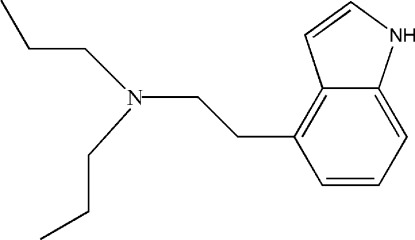


## Experimental

### 

#### Crystal data

C_16_H_24_N_2_
*M* *_r_* = 244.37
Orthorhombic, 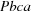

*a* = 12.1100 (9) Å
*b* = 15.5996 (12) Å
*c* = 16.0253 (12) Å
*V* = 3027.4 (4) Å^3^
*Z* = 8
Mo *K*α radiation
μ = 0.06 mm^−1^
*T* = 293 K
0.30 × 0.20 × 0.10 mm

#### Data collection

Enraf–Nonius CAD-4 diffractometer
Absorption correction: ψ scan (North *et al.*, 1968 ▶) *T*_min_ = 0.981, *T*_max_ = 0.994
2757 measured reflections
2757 independent reflections
1294 reflections with *I* > 2σ(*I*)
3 standard reflections every 200 reflections intensity decay: 1%

#### Refinement

*R*[*F* ^2^ > 2σ(*F* ^2^)] = 0.060
*wR*(*F* ^2^) = 0.122
*S* = 1.00
2757 reflections
163 parameters
2 restraints
H-atom parameters constrained
Δρ_max_ = 0.22 e Å^−3^
Δρ_min_ = −0.12 e Å^−3^

### 

Data collection: *CAD-4 EXPRESS* (Enraf–Nonius, 1994 ▶); cell refinement: *CAD-4 EXPRESS*; data reduction: *XCAD4* (Harms & Wocadlo, 1995 ▶); program(s) used to solve structure: *SHELXS97* (Sheldrick, 2008 ▶); program(s) used to refine structure: *SHELXL97* (Sheldrick, 2008 ▶); molecular graphics: *SHELXTL* (Sheldrick, 2008 ▶); software used to prepare material for publication: *SHELXTL*.

## Supplementary Material

Crystal structure: contains datablock(s) I, global. DOI: 10.1107/S1600536812015474/ld2053sup1.cif

Structure factors: contains datablock(s) I. DOI: 10.1107/S1600536812015474/ld2053Isup2.hkl

Supplementary material file. DOI: 10.1107/S1600536812015474/ld2053Isup3.cml

Additional supplementary materials:  crystallographic information; 3D view; checkCIF report

## Figures and Tables

**Table 1:** Hydrogen-bond geometry (Å, °)

*D*—H⋯*A*	*D*—H	H⋯*A*	*D*⋯*A*	*D*—H⋯*A*
N1—H1*A*⋯N2^i^	0.86	2.10	2.945 (3)	166

Symmetry code: (i) 
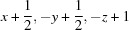
.

**Fig. 1.**
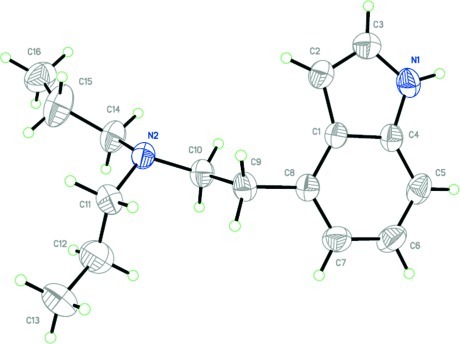
The structure of the title compound, showing the atomic numbering scheme. Non-H atoms are shown with 30% probability displacement ellipsoids.

## supplementary crystallographic information

### Comment

The title compound is an important organic intermediate for the synthesis of 4-[2-(dipropylamino)ethyl]indol-2-one, an important compound for stimulating presynaptic dopamine autoreceptors in mammals (Srivastava *et al.*, 1999). In the process of synthesis, we obtained the crystal of the intermediate and we report its crystal structure.

### Experimental

The title compound was synthesized according to the published procedure (Srivastava *et al.*, 1999). A mixture of 4-[(2-amino)ethyl]indole (3.0 g, 18.72 mmol), 1-bromopropane (4.61 g, 37.44 mmol), K_2_CO_3_ (1 g, 7.25 mmol) in toluene (40 ml) was stirred at reflux temperature until the reaction completion is checked by thin layer silica gel (60–80 mesh) plates. When the mixture reached room temperature, it was filtered and the filtrate was poured in water and extracted with chloroform. The combined extract is washed with 10% brine solution, dried over Na_2_S0_4_ and concentrated to afford the crude product, which was filtered through column. The obtained material was distilled to afford the pure product 3.62 g with a 79% yield. The product (0.3 g) was crystallized from methanol (15 ml) at room temperature to give colorless crystals that were used for data collection.

### Refinement

Carbon- and nitrogen-bound H atoms were placed in calculated positions and were treated as riding on the parent C and N atoms with C—H = 0.96 (methyl), 0.97 (methylene) and N—H = 0.86 Å, *U*_iso_(H) = 1.2 or 1.5 *U*_eq_(C,*N*). The hydrogen atoms for methyl groups were placed in staggered positions. Rigid body restrains were applied to atoms N2, C11, C12 and C13 because of their excessive thermal motion

### Crystal data

**Table:**

C_16_H_24_N_2_	*F*(000) = 1072
*M**_r_* = 244.37	*D*_x_ = 1.072 Mg m^−^^3^
Orthorhombic, *P**b**c**a*	Mo *K*α radiation, λ = 0.71073 Å
Hall symbol: -P 2ac 2ab	Cell parameters from 25 reflections
*a* = 12.1100 (9) Å	θ = 9–13°
*b* = 15.5996 (12) Å	µ = 0.06 mm^−^^1^
*c* = 16.0253 (12) Å	*T* = 293 K
*V* = 3027.4 (4) Å^3^	Block, white
*Z* = 8	0.30 × 0.20 × 0.10 mm

### Data collection

**Table:**

Enraf–Nonius CAD-4 diffractometer	1294 reflections with *I* > 2σ(*I*)
Radiation source: fine-focus sealed tube	*R*_int_ = 0.000
Graphite monochromator	θ_max_ = 25.4°, θ_min_ = 2.5°
ω/2θ scans	*h* = 0→14
Absorption correction: ψ scan (North et al., 1968)	*k* = 0→18
*T*_min_ = 0.981, *T*_max_ = 0.994	*l* = 0→19
2757 measured reflections	3 standard reflections every 200 reflections
2757 independent reflections	intensity decay: 1%

### Refinement

**Table:**

Refinement on *F*^2^	Primary atom site location: structure-invariant direct methods
Least-squares matrix: full	Secondary atom site location: difference Fourier map
*R*[*F*^2^ > 2σ(*F*^2^)] = 0.060	Hydrogen site location: inferred from neighbouring sites
*wR*(*F*^2^) = 0.122	H-atom parameters constrained
*S* = 1.00	*w* = 1/[σ^2^(*F*_o_^2^) + (0.039*P*)^2^] where *P* = (*F*_o_^2^ + 2*F*_c_^2^)/3
2757 reflections	(Δ/σ)_max_ < 0.001
163 parameters	Δρ_max_ = 0.22 e Å^−^^3^
2 restraints	Δρ_min_ = −0.12 e Å^−^^3^

### Special details

**Table:**

Geometry. All e.s.d.'s (except the e.s.d. in the dihedral angle between two l.s. planes) are estimated using the full covariance matrix. The cell e.s.d.'s are taken into account individually in the estimation of e.s.d.'s in distances, angles and torsion angles; correlations between e.s.d.'s in cell parameters are only used when they are defined by crystal symmetry. An approximate (isotropic) treatment of cell e.s.d.'s is used for estimating e.s.d.'s involving l.s. planes.
Refinement. Refinement of *F*^2^ against ALL reflections. The weighted *R*-factor *wR* and goodness of fit *S* are based on *F*^2^, conventional *R*-factors *R* are based on *F*, with *F* set to zero for negative *F*^2^. The threshold expression of *F*^2^ > σ(*F*^2^) is used only for calculating *R*-factors(gt) *etc*. and is not relevant to the choice of reflections for refinement. *R*-factors based on *F*^2^ are statistically about twice as large as those based on *F*, and *R*- factors based on ALL data will be even larger.

### Fractional atomic coordinates and isotropic or equivalent isotropic displacement parameters (Å^2^)

**Table:**

	*x*	*y*	*z*	*U*_iso_*/*U*_eq_	
C1	0.69898 (17)	0.17493 (14)	0.51171 (13)	0.0529 (6)	
N1	0.80610 (17)	0.26448 (12)	0.58576 (12)	0.0672 (6)	
H1A	0.8600	0.2852	0.6144	0.081*	
N2	0.46143 (14)	0.14524 (11)	0.30351 (11)	0.0570 (5)	
C2	0.64447 (19)	0.25584 (16)	0.51942 (15)	0.0661 (7)	
H2A	0.5756	0.2705	0.4981	0.079*	
C3	0.7130 (2)	0.30703 (15)	0.56402 (16)	0.0719 (7)	
H3A	0.6980	0.3638	0.5778	0.086*	
C4	0.79988 (18)	0.18272 (15)	0.55438 (14)	0.0572 (6)	
C5	0.87487 (19)	0.11580 (17)	0.55988 (17)	0.0738 (8)	
H5A	0.9418	0.1220	0.5877	0.089*	
C6	0.8458 (2)	0.04041 (16)	0.52256 (17)	0.0809 (8)	
H6A	0.8942	−0.0058	0.5252	0.097*	
C7	0.7459 (2)	0.03062 (15)	0.48071 (16)	0.0736 (7)	
H7A	0.7294	−0.0219	0.4563	0.088*	
C8	0.67057 (17)	0.09681 (14)	0.47445 (13)	0.0562 (6)	
C9	0.56340 (17)	0.08773 (14)	0.42753 (14)	0.0611 (6)	
H9A	0.5043	0.1133	0.4599	0.073*	
H9B	0.5467	0.0274	0.4204	0.073*	
C10	0.56870 (18)	0.13063 (14)	0.34234 (13)	0.0608 (6)	
H10A	0.6061	0.1853	0.3482	0.073*	
H10B	0.6128	0.0952	0.3054	0.073*	
C11	0.3925 (2)	0.06598 (15)	0.29295 (17)	0.0822 (8)	
H11A	0.3830	0.0401	0.3475	0.099*	
H11B	0.3200	0.0835	0.2737	0.099*	
C12	0.4330 (2)	0.00010 (17)	0.2365 (2)	0.1074 (10)	
H12A	0.5053	−0.0195	0.2543	0.129*	
H12B	0.4396	0.0231	0.1804	0.129*	
C13	0.3492 (3)	−0.07629 (16)	0.2374 (2)	0.1313 (13)	
H13A	0.3739	−0.1201	0.1996	0.197*	
H13B	0.2777	−0.0562	0.2203	0.197*	
H13C	0.3445	−0.0994	0.2928	0.197*	
C14	0.4724 (2)	0.19861 (16)	0.22911 (15)	0.0805 (8)	
H14A	0.5079	0.1647	0.1860	0.097*	
H14B	0.5215	0.2459	0.2423	0.097*	
C15	0.3695 (2)	0.2346 (2)	0.1942 (2)	0.1199 (11)	
H15A	0.3244	0.2555	0.2398	0.144*	
H15B	0.3289	0.1888	0.1670	0.144*	
C16	0.3850 (2)	0.30558 (17)	0.13303 (18)	0.1030 (10)	
H16A	0.3141	0.3258	0.1146	0.155*	
H16B	0.4262	0.2850	0.0860	0.155*	
H16C	0.4245	0.3517	0.1591	0.155*	

### Atomic displacement parameters (Å^2^)

**Table:**

	*U*^11^	*U*^22^	*U*^33^	*U*^12^	*U*^13^	*U*^23^
C1	0.0514 (12)	0.0586 (14)	0.0488 (14)	−0.0011 (12)	−0.0021 (12)	0.0050 (12)
N1	0.0656 (13)	0.0724 (14)	0.0634 (14)	−0.0148 (11)	−0.0121 (11)	0.0002 (11)
N2	0.0528 (11)	0.0663 (11)	0.0520 (12)	0.0045 (9)	−0.0036 (10)	0.0009 (10)
C2	0.0560 (14)	0.0747 (16)	0.0675 (18)	0.0034 (13)	−0.0038 (14)	0.0044 (15)
C3	0.0754 (17)	0.0649 (16)	0.0755 (19)	0.0011 (15)	−0.0004 (15)	−0.0017 (15)
C4	0.0540 (14)	0.0626 (16)	0.0549 (15)	−0.0124 (13)	−0.0051 (13)	0.0077 (13)
C5	0.0543 (15)	0.0775 (18)	0.090 (2)	−0.0078 (14)	−0.0185 (14)	0.0184 (16)
C6	0.0620 (17)	0.0649 (17)	0.116 (2)	0.0074 (13)	−0.0148 (17)	0.0109 (17)
C7	0.0730 (17)	0.0570 (15)	0.091 (2)	−0.0010 (14)	−0.0053 (16)	−0.0016 (14)
C8	0.0528 (14)	0.0616 (15)	0.0542 (15)	−0.0063 (12)	−0.0022 (12)	0.0056 (13)
C9	0.0570 (14)	0.0686 (15)	0.0575 (15)	−0.0113 (12)	−0.0067 (13)	0.0029 (13)
C10	0.0523 (14)	0.0735 (16)	0.0567 (16)	−0.0031 (12)	0.0003 (13)	0.0031 (13)
C11	0.0859 (19)	0.0750 (16)	0.086 (2)	−0.0045 (12)	−0.0250 (16)	−0.0099 (16)
C12	0.110 (2)	0.098 (2)	0.115 (3)	−0.0031 (16)	−0.004 (2)	−0.015 (2)
C13	0.142 (3)	0.086 (2)	0.166 (3)	−0.0158 (17)	−0.045 (2)	−0.032 (2)
C14	0.0766 (18)	0.099 (2)	0.065 (2)	0.0077 (15)	−0.0086 (15)	0.0167 (16)
C15	0.090 (2)	0.170 (3)	0.100 (3)	0.013 (2)	−0.010 (2)	0.046 (2)
C16	0.125 (2)	0.101 (2)	0.083 (2)	0.0286 (19)	−0.0048 (19)	0.0154 (19)

### Geometric parameters (Å, º)

**Table:**

C1—C8	1.400 (3)	C9—H9B	0.9700
C1—C4	1.405 (3)	C10—H10A	0.9700
C1—C2	1.430 (3)	C10—H10B	0.9700
N1—C3	1.354 (3)	C11—C12	1.455 (3)
N1—C4	1.373 (2)	C11—H11A	0.9700
N1—H1A	0.8600	C11—H11B	0.9700
N2—C10	1.458 (2)	C12—C13	1.566 (3)
N2—C14	1.460 (3)	C12—H12A	0.9700
N2—C11	1.501 (2)	C12—H12B	0.9700
C2—C3	1.355 (3)	C13—H13A	0.9600
C2—H2A	0.9300	C13—H13B	0.9600
C3—H3A	0.9300	C13—H13C	0.9600
C4—C5	1.386 (3)	C14—C15	1.477 (3)
C5—C6	1.366 (3)	C14—H14A	0.9700
C5—H5A	0.9300	C14—H14B	0.9700
C6—C7	1.391 (3)	C15—C16	1.490 (3)
C6—H6A	0.9300	C15—H15A	0.9700
C7—C8	1.381 (3)	C15—H15B	0.9700
C7—H7A	0.9300	C16—H16A	0.9600
C8—C9	1.507 (3)	C16—H16B	0.9600
C9—C10	1.522 (3)	C16—H16C	0.9600
C9—H9A	0.9700		
			
C8—C1—C4	119.8 (2)	N2—C10—H10B	108.6
C8—C1—C2	133.8 (2)	C9—C10—H10B	108.6
C4—C1—C2	106.4 (2)	H10A—C10—H10B	107.6
C3—N1—C4	108.38 (19)	C12—C11—N2	117.7 (2)
C3—N1—H1A	125.8	C12—C11—H11A	107.9
C4—N1—H1A	125.8	N2—C11—H11A	107.9
C10—N2—C14	110.90 (17)	C12—C11—H11B	107.9
C10—N2—C11	114.50 (17)	N2—C11—H11B	107.9
C14—N2—C11	115.34 (19)	H11A—C11—H11B	107.2
C3—C2—C1	106.4 (2)	C11—C12—C13	108.2 (2)
C3—C2—H2A	126.8	C11—C12—H12A	110.0
C1—C2—H2A	126.8	C13—C12—H12A	110.0
N1—C3—C2	110.9 (2)	C11—C12—H12B	110.0
N1—C3—H3A	124.6	C13—C12—H12B	110.0
C2—C3—H3A	124.6	H12A—C12—H12B	108.4
N1—C4—C5	129.8 (2)	C12—C13—H13A	109.5
N1—C4—C1	107.8 (2)	C12—C13—H13B	109.5
C5—C4—C1	122.4 (2)	H13A—C13—H13B	109.5
C6—C5—C4	116.8 (2)	C12—C13—H13C	109.5
C6—C5—H5A	121.6	H13A—C13—H13C	109.5
C4—C5—H5A	121.6	H13B—C13—H13C	109.5
C5—C6—C7	122.0 (2)	N2—C14—C15	116.7 (2)
C5—C6—H6A	119.0	N2—C14—H14A	108.1
C7—C6—H6A	119.0	C15—C14—H14A	108.1
C8—C7—C6	121.8 (2)	N2—C14—H14B	108.1
C8—C7—H7A	119.1	C15—C14—H14B	108.1
C6—C7—H7A	119.1	H14A—C14—H14B	107.3
C7—C8—C1	117.2 (2)	C14—C15—C16	115.2 (3)
C7—C8—C9	122.3 (2)	C14—C15—H15A	108.5
C1—C8—C9	120.4 (2)	C16—C15—H15A	108.5
C8—C9—C10	111.72 (17)	C14—C15—H15B	108.5
C8—C9—H9A	109.3	C16—C15—H15B	108.5
C10—C9—H9A	109.3	H15A—C15—H15B	107.5
C8—C9—H9B	109.3	C15—C16—H16A	109.5
C10—C9—H9B	109.3	C15—C16—H16B	109.5
H9A—C9—H9B	107.9	H16A—C16—H16B	109.5
N2—C10—C9	114.46 (18)	C15—C16—H16C	109.5
N2—C10—H10A	108.6	H16A—C16—H16C	109.5
C9—C10—H10A	108.6	H16B—C16—H16C	109.5
			
C8—C1—C2—C3	178.9 (2)	C4—C1—C8—C7	−1.0 (3)
C4—C1—C2—C3	0.6 (2)	C2—C1—C8—C7	−179.2 (2)
C4—N1—C3—C2	0.4 (3)	C4—C1—C8—C9	−179.20 (19)
C1—C2—C3—N1	−0.6 (3)	C2—C1—C8—C9	2.7 (4)
C3—N1—C4—C5	179.7 (2)	C7—C8—C9—C10	−102.4 (2)
C3—N1—C4—C1	0.0 (2)	C1—C8—C9—C10	75.7 (3)
C8—C1—C4—N1	−178.98 (19)	C14—N2—C10—C9	170.78 (18)
C2—C1—C4—N1	−0.4 (2)	C11—N2—C10—C9	−56.6 (3)
C8—C1—C4—C5	1.4 (3)	C8—C9—C10—N2	−163.88 (18)
C2—C1—C4—C5	180.0 (2)	C10—N2—C11—C12	−65.2 (3)
N1—C4—C5—C6	179.5 (2)	C14—N2—C11—C12	65.3 (3)
C1—C4—C5—C6	−0.9 (4)	N2—C11—C12—C13	178.8 (2)
C4—C5—C6—C7	0.1 (4)	C10—N2—C14—C15	−166.8 (2)
C5—C6—C7—C8	0.1 (4)	C11—N2—C14—C15	61.0 (3)
C6—C7—C8—C1	0.3 (4)	N2—C14—C15—C16	164.9 (2)
C6—C7—C8—C9	178.5 (2)		

### Hydrogen-bond geometry (Å, º)

**Table:**

*D*—H···*A*	*D*—H	H···*A*	*D*···*A*	*D*—H···*A*
N1—H1*A*···N2^i^	0.86	2.10	2.945 (3)	166

Symmetry code: (i) *x*+1/2, −*y*+1/2, −*z*+1.
